# Accelerometer-Based Event Detector for Low-Power Applications

**DOI:** 10.3390/s131013978

**Published:** 2013-10-16

**Authors:** József Smidla, Gyula Simon

**Affiliations:** Department of Computer Science and Systems Technology, University of Pannonia, Veszprém H-8200, Hungary; E-Mail: simon@dcs.uni-pannon.hu

**Keywords:** accelerometer, autocovariance, intelligent signal processing, measurement instrumentation, power efficiency, MEMS

## Abstract

In this paper, an adaptive, autocovariance-based event detection algorithm is proposed, which can be used with micro-electro-mechanical systems (MEMS) accelerometer sensors to build inexpensive and power efficient event detectors. The algorithm works well with low signal-to-noise ratio input signals, and its computational complexity is very low, allowing its utilization on inexpensive low-end embedded sensor devices. The proposed algorithm decreases its energy consumption by lowering its duty cycle, as much as the event to be detected allows it. The performance of the algorithm is tested and compared to the conventional filter-based approach. The comparison was performed in an application where illegal entering of vehicles into restricted areas was detected.

## 1. Introduction

Accelerometers are successfully utilized in event detection systems, e.g., fall detection [1] and movement detection and analysis [2,3]. Seismic vibrations, caused by various sources, can also be detected by accelerometers, e.g., in footstep detection and vehicle detection [4]. In this paper, an accelerometer-based sensor for event detection purposes is proposed. In our application, sensors are used to detect unauthorized traffic in areas where normally no traffic is allowed. The protected area is located in a remote forest, where the system must operate autonomously for a long time with high reliability. In such applications, detections are very rare, but the system must be accurate in the sense that all trespassing vehicles must be detected, and the rate of false positive detections must be very low (false detections cause unnecessary and expensive intervention).

In real-world applications, sensors are often deployed in remote, hostile environments, where sensors must operate autonomously, using their limited power supply; thus, the lifetime of the sensors is often of key importance. In such cases, the energy efficiency of the sensor is a key design factor.

Duty cycling is a general concept that is often used to decrease energy consumption; see e.g., [5]. A similar approach is used in the proposed solution: sampling and signal processing is performed only in short time intervals, followed by a low-power state of the sensor. The applicable duty cycle is constrained by the properties of the detected event and the required quality of service.

The proposed solution builds on an enhanced version of the autocovariance-based detection algorithm [6]. The enhanced signal processing algorithm has extremely low computational needs; thus, it can be implemented on devices with scarce resources and, also, allows low-power operation for the sensor device. The performance of the proposed algorithm will be evaluated using real measurements.

In Section II, related work is reviewed. Section III briefly reviews the algorithm in [6]. The proposed solution will be introduced in Section IV and will be evaluated and compared to other solutions, using real measurements, in Section V. Section VI concludes the paper.

## 2. Related Work

For vehicle detection, several sensory systems are in use. For traffic monitoring in urban environments, two approaches exist: intrusive and non-intrusive sensors. Intrusive sensors require stripping of roads, while this is unnecessary when non-intrusive sensors are used. Intrusive sensors include inductive loops, magnetometers, microloop probes, pneumatic road tubes, piezoelectric cables and weight-in-motion sensors, while non-intrusive sensors include video image processing, microwave radar, laser radar, passive infrared, ultrasonic and passive acoustic arrays. However, most of these solutions are energy demanding and expensive; the deployment is cumbersome, and only a few of them can be used in a concealed application (see [7] for a comprehensive review).

Accelerometers are used in many application areas (e.g., structure monitoring [8], body movement sensing [2,3,9] and event detection [10]) and are also used for vehicle detection and traffic monitoring purposes. In [11], the arrival of trains in railway stations was detected using accelerometers, while in [4], accelerometers were used to monitor traffic.

Micro-electro-mechanical systems (MEMS) are extensively used in a wide range of applications. MEMS accelerometers are one of the most common types of MEMS sensors, due to their simplicity, ease of fabrication, low price and good usability [12]. In MEMS accelerometers, the movement of a seismic mass, attached to a cantilever beam, is detected using capacitive sensing. The damping is provided by the sealed gas around the seismic mass, which also causes significant noise in these devices, due to Brownian noise [13].

Energy efficiency is a key design factor in sensor networking applications where power supply is limited. The research for power-efficient sensors resulted in several hardware solutions, new medium-access protocols and routing methods. In the context of measurement and detection, the Compressive Sensing Theory was invented.

The Theory of Compressive Sensing allows the creation of more efficient sensors by reducing the amount of sampled, processed and transmitted data. Compressive Sensing performs sampling, compression and reconstruction of sparse signals with a smaller number of samples than the Nyquist rate [14]. The same theory has been applied for detection purposes, where the number of measurements required for detection was reduced ([15,16]). Although the Theory of Compressive Sensing is appealing, its application for our purposes is not practical, because of its high computational complexity and the limitations in sampling of the physical sensor (e.g., most sensors can be programmed to perform periodic sampling only).

Duty cycling is widely used in embedded systems: low duty cycle operation of sensors allows the reduction of energy consumption. Duty cycling, however, has challenging aspects when the goal is detection: if the duty cycle is not properly chosen, then the sensor may completely miss an event, and the sleeping nodes may increase the reaction time.

In [17], a probabilistic scheduling of duty cycling was proposed; thus, a balance between the sensors' lifetime and the quality of service was provided. In [18], the detection performance, as a function of duty cycling, was examined, and a wakeup process was proposed. In [19], a control mechanism was proposed, which changes the duty cycle, based on the detected event properties.

In [6], an accelerometer-based detector was proposed, which uses the autocovariance of the signal. In this paper, this method will be enhanced, and a duty-cycle mechanism will be applied to the basic detection scheme, to provide both low-energy consumption and low computational complexity.

## 3. Autocovariance-Based Event Detection

The solution proposed in [6] is based on the autocovariance of the signal. Let *x*_Δ_ denote the shifted version of *x*, such that *x*_Δ_(*k*) = *x*(*k* – Δ), and *E*[.] is the expected value operator. The autocovariance of *x* is defined as follows:

(1) $$C_{x,x}(\Delta )=E[(x-E\left[x\right])(x_{\Delta }-E\left[x_{\Delta }\right]$$

The autocovariance *C_x,x_*(Δ) can be computed in the following way:

(2) $$C_{x.x}(\Delta )=E\left[xx_{\Delta }\right]-E\left[x\right]E\left[x_{\Delta }\right]$$

**Figure 1.**
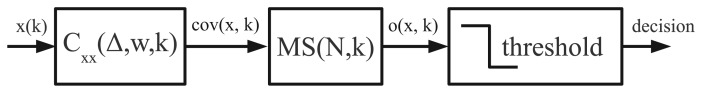
Flow chart of the algorithm in [6].

The computed *C_x,x_*(Δ) is low for ∀Δ ≠ 0 in the case when *x* is white background noise. However, an event in *x* results in a higher *C_x_*_,_*_x_*(Δ) for a wide range of Δ values. Based on this assumption, a detector was proposed in [6]. As Figure 1 shows, The final decision is based on the estimated *o*(x, *k*) mean square of the autocovariance *cov*(*k*) of signal *x*(*k*): when *o*(x, *k*) is higher than a fixed threshold level, the algorithm raises an alarm. The detailed operation is the following:

Let x be a vector and x[] be a subvector containing (*x*(*a*)*,x*(*a* + 1),…, *x*(*b*)), where *a* < *b*. The estimate of *E*[x] is computed from x[] as follows:

(3) $$\hat{E}[x[a:b]]=\frac{1}{b-a+1}\sum_{k=a}^{b}x(k)$$

*E*[*xx*_Δ_] is approximated by *Ê*[x[*a* : *b*]*^T^*x_Δ_[*a* : *b*]] as follows:

(4) $$\hat{E}[{\text{x}[a:b]}^{T}{\text{x}}_{\Delta }[a:b]]=\frac{\sum_{k=a}^{b}x(k)x_{\Delta }(k)}{b-a+1}$$

Based on Equations (3) and (4), the autocovariance in Equation (2) is approximated in the following way:

(5) $$C_{x,x}(\Delta ,w,k)=\hat{E}\left[{\text{x}}_{1}^{T}{\text{x}}_{2}\right]-\hat{E}[{\text{x}}_{1}]\hat{E}[{\text{x}}_{2}]$$

where *w* denotes the window size, x_1_ = x[*k* − *w* + 1 : *k*] and x_2_ = x_Δ_ [*k* − *w* + 1 : *k*].

The output o(x, *k*) of the algorithm is the mean square of the last *M* autocovariance values:

(6) $$\text{o}(\text{x},k)=\frac{1}{M}\sum_{j=k-M+1}^{k}C_{x,x}{\left(\Delta ,w,j\right)}^{2}$$

In [6] *w* = *M* = 256, Δ = 1 and sampling frequency *f_s_* = 300 Hz was used. The computational need of the algorithm is extremely low: the algorithm performs six subtractions, eight additions, five multiplications and four-bit shifts per sample.

The solution proposed in [6] requires the continuous operation of the sensor: sampling and processing is performed with a fixed sampling frequency. In sensors with limited power supply, the energy efficiency is often provided by the duty cycling of the operation, *i.e.*, sleeping and operating states are periodically alternated. In our proposed solution, a similar approach will be used: the method proposed in [6] will be extended to handle periodic block-wise operation.

## 4. Proposed Solution

**Figure 2.**
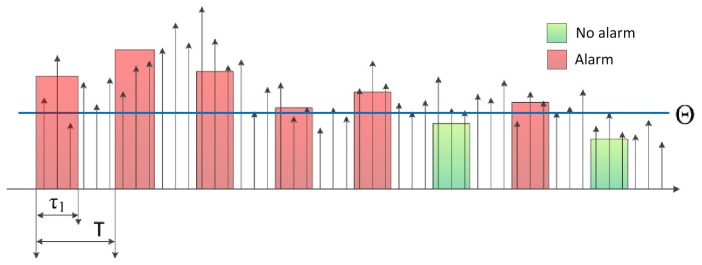
Operation of the Block-wise Autocovariance-based Algorithm (BAC) algorithm. Arrows represent acceleration data. Rectangles show segments where sampling and processing is performed; red and green rectangles represent segments where the computed autocovariance is higher and lower than the threshold, respectively.

Instead of continuous sampling and processing, in the proposed solution, the sensor is switched on periodically, with a period of *T*, for a short time interval *τ*_1_. While the sensor is on, a block of samples is collected and processed. After sampling and processing of the block, the sensor returns to sleep mode, as shown in Figure 2.

In this section, three variants of the algorithm will be proposed: the simple block-wise algorithm, a block-wise algorithm with an additional evaluation phase and, finally, its adaptive version.

### 4.1. Block-Wise Autocovariance-BasedAlgorithm (BAC)

The algorithm is illustrated in Figure 2. With a period of *T*, the sensor is switched on, and the output of the accelerometer is collected for *τ*_1_ time. From the collected *n*_1_ = ⌊*τ*_1_*f_s_*⌋ samples in record *x*, the autocovariance estimate *Ĉ_x,x_* at Δ = 1 is computed for the last active period as follows:

(7) $${\hat{C}}_{x,x}(x)=\frac{1}{n-1}{\sum_{j=1}^{n-1}x(j)x(j+1)-\left(\frac{1}{n-1}\sum_{j=1}^{n-1}x(j)\right)}^{2}$$

Based on this (single) autocovariance value, the decision is made, using a threshold Θ:

(8) $${\text{alarm}}_{\text{BAC}}=\{\begin{array}{rr} \text{on}, & \text{if}\;{\hat{C}}_{x,x}(x)>\Theta \\ \text{off}, & \text{otherwise} \end{array}$$

The pseudo code of the BAC algorithm is shown in Appendix A.

An application specific constraint on *T* is the length of the perturbation caused by the vehicles. If *T* is set larger than the length of the detectable perturbation, then the vehicle may pass the sensor between two measurements undetected.

To conserve energy, the length *τ*_1_ of active periods should be short. However, due to the short data segments, the *Ĉ_x,x_* estimates have large variance; thus, events occasionally may produce low *Ĉ_x,x_* values instead of the expected high values; similarly, background noise occasionally may produce unexpectedly high *Ĉ_x,x_* values. To avoid false negatives (*i.e.*, missed events), threshold Θ should be low, and in order to avoid false positives (*i.e.*, false alarms when no event is present), Θ should be set high. The next variant of the algorithm eliminates this problem.

### 4.2. Block-Wise Autocovariance-Based Algorithm With Validation (BACV)

The Block-wise Autocovariance-based Algorithm With Validation (BACV) switches on the sensor with period *T*, when it collects samples for time interval of length *τ*_1_ and computes *Ĉ_x,x_* by Equation (7), similarly to BAC. A second round of validation is triggered if the autocovariance estimate *Ĉ_x,x_* is larger than a threshold *ϑ*:

(9) $$\text{trigger}=\{\begin{array}{rr} 1, & \text{if}{\hat{C}}_{x,x}>\vartheta \\ 0, & \text{otherwise} \end{array}$$

**Figure 3.**
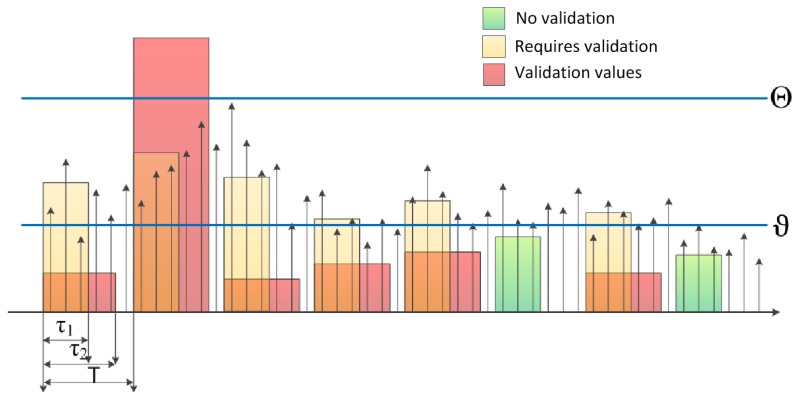
Operation of the Block-wise Autocovariance-based Algorithm With Validation (BACV). Narrow rectangles of width *τ*_1_ represent segments where preliminary sampling and processing is performed; yellow and green rectangles represent segments where the computed autocovariance is higher and lower than the preliminary threshold *ϑ*, respectively Wider rectangles of width *τ*_2_ represent validation phases, the output of which is compared to threshold Θ to produce the detection signal.

If *trigger* = 1, a validation round is started, where a longer data record is used, with a length of *τ*_2_ > *τ*_1_, as illustrated in Figure 3. If the autocovariance estimate computed in the validation phase is larger than Θ, then an alarm is emitted. In the BACV algorithm threshold, *ϑ* can be set low enough to avoid false negatives. The longer validation phase produces results with decreased variance; thus, Θ can be set higher to avoid false negative detections. Note that in the preliminary checking phase, only one autocovariance value is computed (see Equation (7)), while in the validation round, the algorithm computes the mean square autocovariance estimates using a sliding window of a length of *w*:

(10) $$c(k)=\frac{1}{w}\sum_{j=k}^{k+w-1}{\left({\hat{C}}_{x,x}(j:j+w-1)\right)}^{2}$$

where *k* = 1, 2,…, *n*_2_ − *w* and *n*_2_ = ⌊*τ*_2_*f_s_* ⌋is the length of the validation record. Note that *c*(*k*) can be computed very efficiently using the method described in [6]. The decision is made by comparing the maximal value of *c*(*k*) to the threshold Θ, as follows:

(11) $${\text{alarm}}_{\text{BACV}}=\{\begin{array}{ll} \text{on}, & \text{if}\;\max_{k}(c(k))>\Theta \\ \text{off}, & \text{otherwise} \end{array}$$

The pseudo code of BACV is listed in Appendix B.

The BACV algorithm uses constant thresholds, *ϑ* and Θ. However, when noise properties change, the adaptation of the thresholds is necessary The following extension of the algorithm sets the thresholds automatically.

### 4.3. Adaptive BACV Algorithm (ABACV)

**Figure 4.**
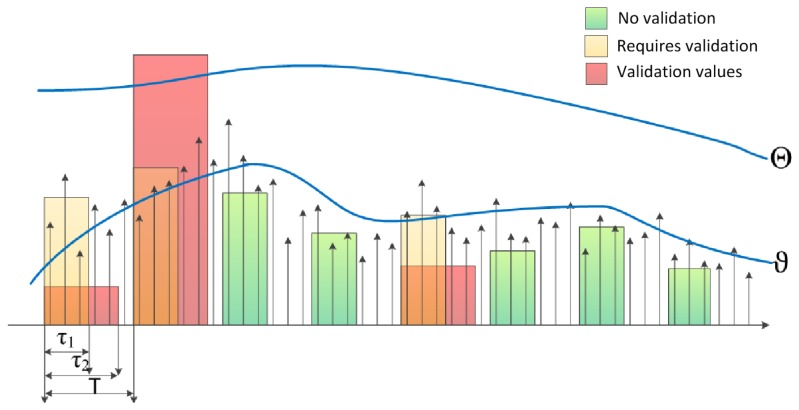
The operation of the Adaptive BACV Algorithm (ABACV). Thresholds *ϑ* and Θ are changed to follow changes over noise properties.

The Adaptive BACV Algorithm (ABACV) uses a similar mechanism to BACV, but adapts the thresholds, *ϑ* and Θ, in each period, as illustrated in Figure 4. The strategy of the control of *ϑ* is the following: set the threshold *ϑ*, so that the rate of triggering of the second validation round is around a constant value, *ζ*. This strategy provides a constant (low) rate of unnecessary validation rounds when no events are present, but at the same time, finely tunes *ϑ*, so that it follows the changes of the noise power. The rate of triggering is computed using exponential averaging, forgetting factor *α*:

(12) $${\text{rate}}_{k+1}:=\alpha {\text{rate}}_{k}+(1-\alpha )\text{trigger}$$

where *trigger* is computed according to Equation (9).

The control mechanism increases the value of *ϑ*, while the triggering rate is higher than *ζ* and decreases *ϑ* otherwise, in small steps *ε*:

(13) ϑ=ϑ+εsignum(rate−ζ)

where the desired rate *ζ* is set in the range of 0.02 … 0.05.

To allow the adaptation of Θ, the average mean square of the noise autocovariance is estimated, using the computed *Ĉ_x,x_* values in each period, as follows:

(14) $$d=\{\begin{array}{ll} \alpha d+(1-\alpha ){\hat{C}}_{x,x}^{2}, & \text{if}\;{\hat{C}}_{x,x}^{2}<\sum \\ \text{d}, & \text{otherwise} \end{array}$$

Note that *d* should estimate the mean square autocovariance of the *noise*, but the high autocovariance of events distort the estimated value. The conditional update in Equation (14) decreases the effect of high *Ĉ_x,x_* values caused by events, but does not affect the noise estimate. The threshold Σ is set to a value significantly higher than the noise autocovariance and smaller than autocovariances measured in the presence of events.

The threshold is adapted as Θ = *λd*, where λ provides a sufficiently large gap between the mean square noise autocovariance and the detection level. In our experiments, the choice of λ = 7..10 produced a reliable operation.

The alarm is raised if the mean square autocovariance estimate is higher than Θ, *i.e.*:

(15) $${\text{alarm}}_{\text{ABACV}}=\{\begin{array}{ll} \text{on}, & \text{if}\;{\text{max}}_{k}(c(k))>\lambda d\\ \text{off}, & \text{otherwise} \end{array}$$

The pseudo code of the ABACV algorithm is shown in Appendix B.

## 5. Evaluation

In this section, the performance of the proposed methods will be analyzed. First, the sensor hardware and the data used in the analysis will be introduced. A robustness metric will be defined, and the algorithms will be analyzed with it. Tests will be introduced to compare the detection capabilities of the algorithms, followed by analysis of the the energy efficiency of the algorithms. Finally, the computational complexity of the proposed methods will be analyzed.

### 5.1. Test Hardware and Test Data

**Figure 5.**
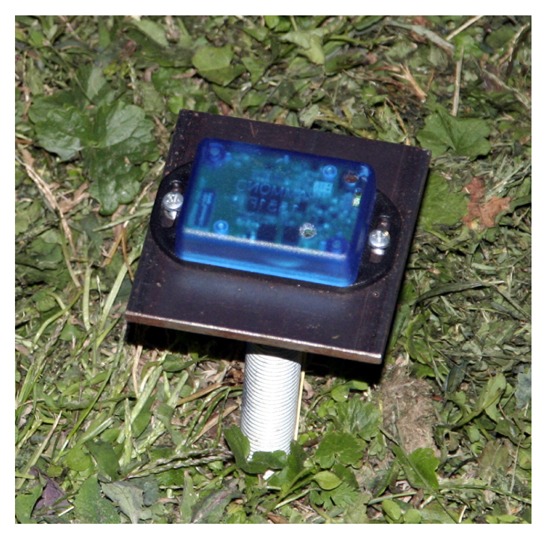
The sensor node.

The test device includes the BMA-180 accelerometer and an eight-bit ATMega128RFA1 processor, running at 16 MHz with 16 kB of RAM and 128 kB of flash memory. It also has an internal EEPROM with a size of 4 kB to store configuration data. The internal radio of the ATMega128RFA1 chip is used to send measurement/detection data in a wireless manner. The device was programmed in nesC under TinyOS [20]. Figure 5 shows the deployed device.

For evaluation purposes, a recording of a length of 23 minutes was made, which contains the raw measurement data obtained from one channel of the accelerometer, using a sampling frequency of *f_s_* = 300 Hz. The sensor was placed 5.6 meters from the road, and during the recording, nine vehicles of different sizes and types were passing by. The recording was annotated by hand, marking sections where a vehicle was present (*V*_1_*, V2*, …, *Vg*) and sections where only background noise was measured.

### 5.2. Robustness Test

The proposed algorithms make their decision by comparing an autocovariance (or mean square autocovariance) estimate to a threshold Θ. The robustness or sensitivity test evaluates how easy it is to select a proper Θ, so that the noise effect remains below Θ, but the effect of the events is higher than Θ. For this purpose, a robustness metric will be defined.

**Figure 6.**
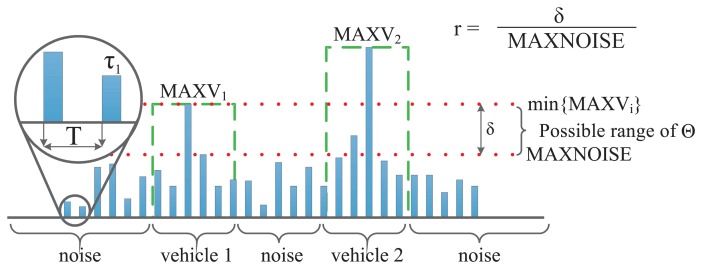
The derivation of the robustness parameter, *r*. The sensor is switched on with period *T* for time *τ*_1_

In each recorded section *V_i_*, the maximum output of the tested algorithm (*MAXV_i_*) was computed, along with the maximum output of the algorithm under the test in the noisy sections (*MAXNOISE*). An important parameter characterizing the robustness of the algorithms is the distance between the effects of the highest noise and the most quiet vehicle, *i.e.*, $\delta =\min_{i=1}^{9}\{{\text{MAXV}}_{i}\}-\text{MAXNOISE}$ (see Figure 6 for an illustration). Obviously, if *δ* ≤ 0, no threshold exists for which the detector is able to detect all the vehicles and does not provide false detections. When *δ* > 0 is small, then it is hard to find a reliable threshold, while with high *δ*, a wide range of thresholds provides reliable operation.

In the tests, the effect of parameters *T* and *τ*_1_ was investigated. While the algorithm [6] is shift-invariant, the proposed algorithm, due to its block-wise nature, may produce different results if the input is shifted (since quite different sets of input samples may be used). To take into consideration the shift-variant nature of the algorithm, several experiments were created by shifting the same input signal. The full record was used for each (*T*, *τ*) pair, and the test was repeated ⌊*Tf_s_*⌋ times, by shifting the record by one sample in each test. Thus, for each (*T*, *τ*) pair ⌊*Tf_s_*⌋, experiments were performed, producing for each experiment values $\delta =\min_{i=1}^{9}\{{\text{MAXV}}_{i}(j)\}-\text{MAXNOISE}(j)(j=1,2,\ldots ,\left\lfloor Tf_{s}\right\rfloor )$. The average of *δ_j_* values is computed as follows:

(16) $$\delta =\frac{1}{\left\lfloor Tf_{s}\right\rfloor }\sum_{j=1}^{\left\lfloor Tf_{s}\right\rfloor }\delta_{j}$$

Note that the values, *MAXV_i_*, (*MAXNOISE*) and *δ*, are computed using all the experiments produced by the record shifting.

The metric, *r*, to characterize robustness is defined as the ratio of *δ* and the maximum noise level, as follows:

(17) $$r=\frac{\delta }{\text{MAXNOISE}}$$

**Figure 7.**
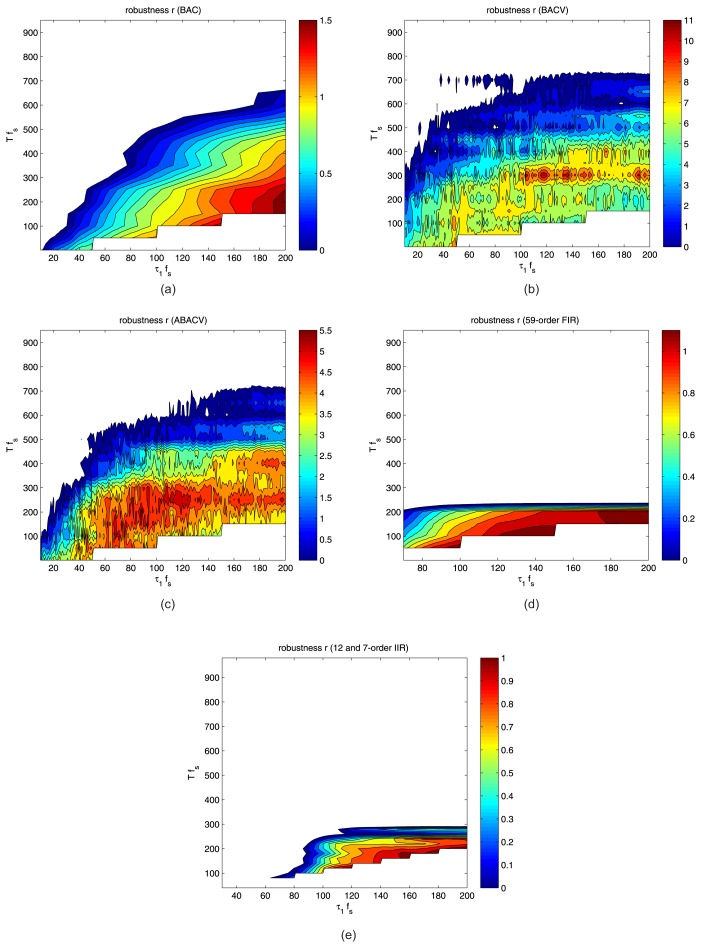
Robustness test of (**a**) BAC, (**b**) BACV, (**c**) ABACV algorithms, (**d**) a bandpass FIR-based algorithm (**e**) and an IIR-based algorithm.

The result of the robustness tests can be seen in Figure 7, which shows the *r* values, calculated with Equation (17). The colored areas of the figures show (*T*, *τ*) pairs, where the *r* is positive; the highest values are represented with red, the smallest values, with blue color. As intuitively expected, *T* must remain small, otherwise *r* becomes negative (*i.e.*, if the sampling intervals are far from each other, an event may completely be lost, depending on the phase). Similarly, larger *τ*_1_ values give better results (since larger records give better estimates). In Figure 7a–c, the results for algorithms BAC, BACV and ABACV are shown, respectively. The parameters of the proposed algorithms during the test were the following: *w* = 128, *ζ* = 0.03, *n_2_* = 400. Clearly BACV and ABACV are more robust than BAC. The robustness value of BAC does not exceed 1.5, while BACV and ABACV reaches *r* = 3 … 5 in a wide range of *T* and *τ*_1_ parameters. Note that BACV has some extremely good values (greater than 10), but only for a very small set of (*τ*_1_*,T*) values. Both BACV and ABACV are very robust for *τ*_1_*f_s_* > 60 and *Tf_s_* < 300.

In Figure 7d, a bandpass FIR (finite impulse response) filter-based solution is shown, and Figure 7e shows an IIR (infinite impulse response) filter-based solution [4]. Both filter-based methods use bandpass filters with a pass band of [*40Hz*–*60Hz*], where the majority of the event power was observed in our measurements. The FIR filter was implemented as a 59-order equiripple filter, while the IIR filter-based solution uses a 12-order elliptic bandpass filter *G*_1_. The IIR-based solution also utilizes a seven-order elliptic low-pass post filter *G*_2_, where the input of the *G*_2_ filter is the square of the output of the *G*_1_ post-filter, as was proposed in [4]. According to Figure 7, the filter-based approaches show inferior robustness properties (their robustness parameter hardly exceed one) and, thus, are more sensitive to the proper choice of parameters *T*, *τ*_1_ and Θ.

### 5.3. Performance Evaluation

In this section, the performance of the proposed algorithms will be evaluated by measuring the rates of false detections as a function of parameter settings. In the test, we measure the false negative and positive ratios, defined as follows: the false negative ratio is the number of missed events over the number of total events present during the test; the false positive ratio is the number of false detections (alarms when no events were present) over the total number of possible non-overlapping events during the test. The latter quantity was estimated as the length of the record divided by the average length of one event.

As in the case of the robustness test, new test cases were generated by shifting the original record: for a period of *T*, ⌊*Tf_s_*⌋, new test records were generated by shifting the record by one sample at a time. The parameter settings were the following: *w* = 128, *ζ* = 0.03, *n*_2_ = 400.

**Figure 8.**
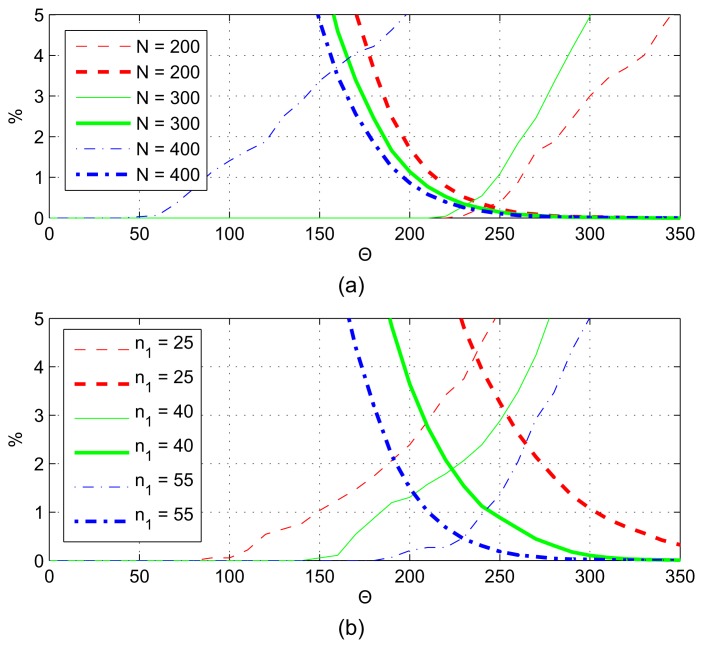
Error rates of BAC, for (**a**) different *n*_1_ and (**b**) *N* values. Thin line: false negative; thick line: false positive.

The results of algorithm BAC are shown in Figure 8, by changing parameters *n*_1_*= τf_s_* and *N* = *Tf_s_*. In Figure 8a, *N* = 230, and in Figure 8b, *n*_1_ = 55. Thin lines represent false negative (FN) decisions, and thick lines represent false positives (FP). Both ratios improve (decrease) when *τ*_1_ is increased or *T* is decreased. According to Figure 8, the cutoff points (where FP = FN) can be as low as 0.4%, for a narrow range of Θ values.

**Figure 9.**
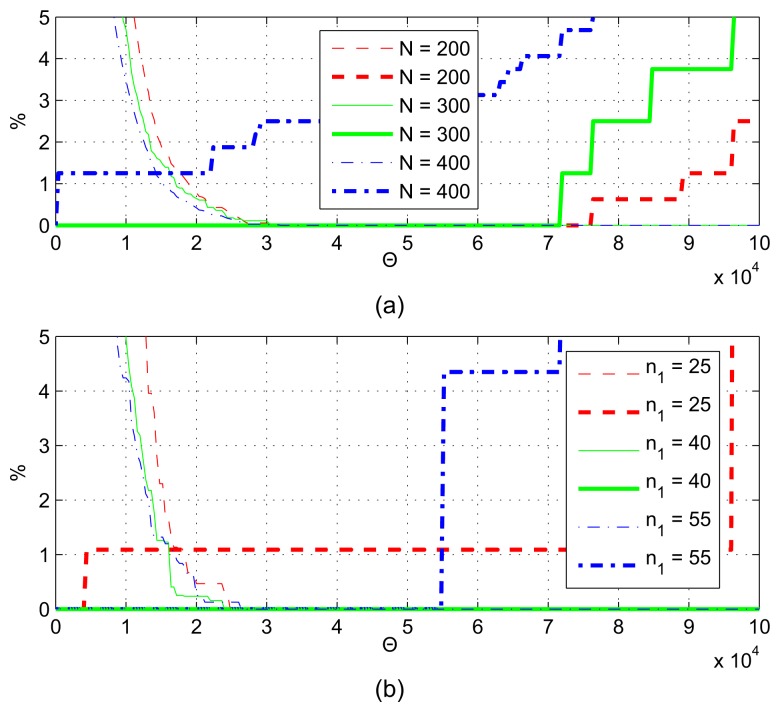
Error rates of BACV, for different (**a**) *n*_1_ and (**b**) *N* values.

The error rates for BACV are shown in Figure 9, with *N* = 230 in Figure 8a and *n*_1_ = 55 in Figure 8b. The error rates can be reduced to zero with *N* ≤ 300 and *n*_1_ ≥ 40, for quite a wide range of Θ values; thus, the BACV algorithm has better performance and is more robust than BAC.

**Figure 10.**
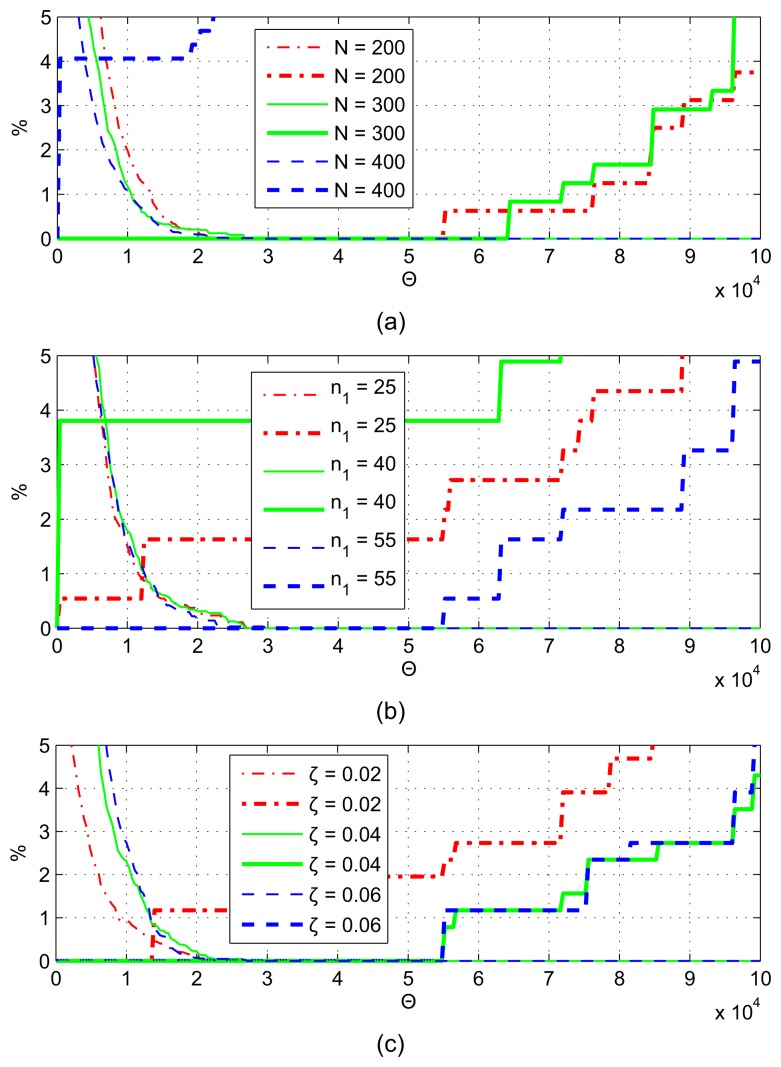
Error rates of ABACV, for different (**a**) *n*_1_, (**b**) *N* and (**c**) *ζ* values.

The error rates for ABACV are shown in Figure 10, for different parameters *N*, *n*_1_ and *ζ*. Note that in order to perform this test, the adaptation of Θ was switched off (but the adaptation of *ϑ* was on). The results are quite similar to that of BACV. Zero error rate can be achieved for a wide range of parameter settings, for a wide range of Θ; thus, the adaptation mechanism has a safe margin of error.

**Figure 11.**
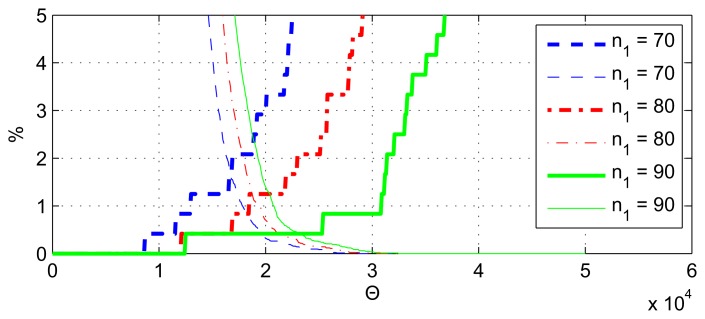
Error rates of the FIR-based algorithm for different *n*_1_ values.

**Figure 12.**
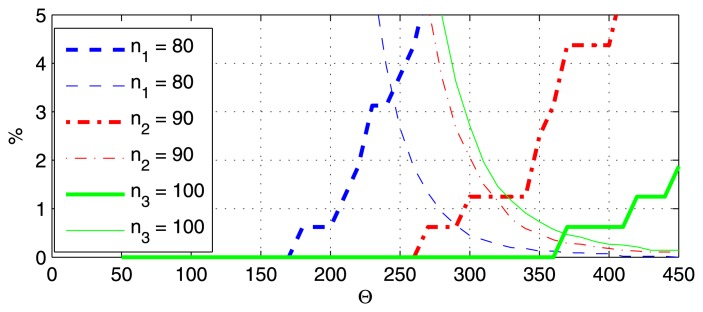
Error rates of the IIR-based algorithm for different *n*_1_ values.

The error rates for the FIR band-pass filter-based algorithm are shown in Figure 11, and the error rates of the IIR filter-based solution [4] can be seen in Figure 12 for the varying of parameter *n*_1_. With the tested parameters, at the best cutoff point, the error rates are around 0.4% for these filter-based algorithms.

### 5.4. Power Efficiency

The power consumption of sensors in low energy (sleep) mode is negligible, compared to the power consumption in the active state. Thus, the power consumption of the sensor is approximately proportional to the time the sensor spends in the active state. The online algorithm [6] is awake 100% of the time (this algorithm is used as the reference), while the proposed methods decrease their duty cycles. In this section, the power efficiency of the algorithms will be analyzed, as a function of parameters *T* and *τ*_1_.

**Figure 13.**
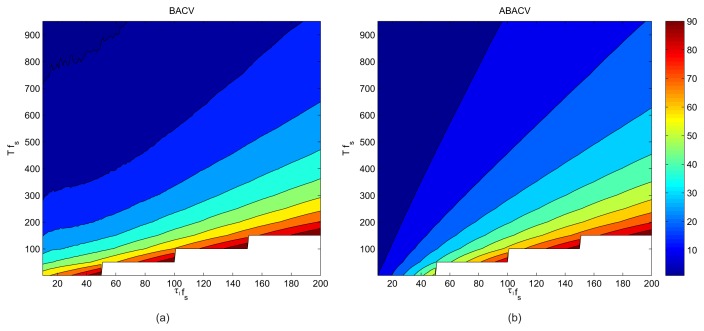
Relative energy consumption of (**a**) BACV and (**b**) ABACV, the energy consumption of the sensor that is awake all the time being 100%.

The power efficiency of the BAC algorithm and the filter-based solutions can be simply derived: the sensor is active for time period *τ*_1_ and is asleep for time period *T* − *τ*; thus, their power efficiency is *τ*_1_/*T*. Algorithms BACV and ABACV, however, occasionally use validation rounds, when the sensor is awake for a longer time. The frequency of the validation rounds depends on other parameters (e.g., *ϑ*, *ζ*) and the input signal. The power efficiencies of BACV and ABACV were measured, using the same recording as in the previous tests. The power consumption, relative to that of the on-line algorithm [6] is shown in Figure 13, *vs. T* and *τ*_1_.

The algorithms are more economic when *T* is large and *τ*_1_ is small, which contradicts the requirements of robustness, as shown in Figure 7. Fortunately, there is a parameter space around *Tf_s_* = [200..300] and *τ*_1_*f_s_* = [40..50], where the algorithm satisfies both requirements. With these parameters, both safe operation with a very low error rate and a good power efficiency (power consumption decreased to 20%) can be provided.

### 5.5. Comparison Tests

The proposed algorithm family and the bandpass filter-based methods were compared, using the same data record as in the previous tests. In this test, all the adaptation mechanisms of the ABACV algorithm were enabled. The results are listed in Table 1. The table shows the relative power consumption of the algorithms (RPC), the false positive (FP) and false negative (FN) rates for various parameter settings. The parameters of the algorithms were varied in the range where relatively good performance was provided.

The performance of the algorithm suggested in [6] was excellent (no false detections), since it uses all the available input data, unlike the proposed algorithms. However, its relative power consumption is 100%.

In the case of BAC, parameters *N*, *n*_1_ and Θ were changed. The algorithm was able to decrease its power consumption rate below 20% and to keep the error rate between 0.19% and 2.38%. In the case of the BACV algorithm, parameters *N*, *n*_1_, *n*_2_ and Θ were varied. The power consumption rate was around 26–37%, and the error rates were close to zero. In the case of ABACV *N*, *n*_1_, *n*_2_, *ζ* and *λ* were varied. The power consumption ratio was around 20%, and the error rate was again close to zero. Note the decrease in power consumption, with respect to BACV; this is due to the successful adaptation of threshold *ϑ*.

For the FIR filter-based algorithm, parameters *N*, *n*_1_, *O* (the order of the FIR filter) and Θ were varied. Finally, *N*, *n_1_* and Θ were varied for the IIR filter-based algorithm. The error rates of these methods are higher, and the power consumption ratio is also higher. Note that the nature of events used in the test determine the possible parameter space and the achievable reduction of power consumption: the length of the signal perturbation caused by a passing by car was around 1–2 s, which is an upper bound for *T*.

### 5.6. Computational Complexity

In this section, the the computational complexities of the algorithms are derived. The BAC algorithm requires *n*_1_ multiplications, 2*n*_1_ additions and two-bit shifts in each *T* time period. For the same amount of data, BACV requires *n*_1_ + 5*p*(*n*_2_ − *n*_1_) multiplications, 2*n*_1_ + 8*p*(*n*_2_ − *n*_1_) additions and 2 + 4*p*(*n*_2_ − *n*_1_)-bit shifts, where *p* is the probability of triggering a validation round. The ABACV algorithm requires n_1_ + 5*ζ*(n2 − n_1_) + 4 multiplications, 2*n*_1_ + 8*ζ*(*n*_2_ − *n*_1_) + 2 additions and 2 + 4ζ(*n*_2_ − *n*_1_)-bit shift operations. The FIR filter-based solution needs *n*_1_*O* multiplications and additions. The IIR-based solution introduced in [4] uses 12- and seven-order IIR elliptical filters (implemented in Transposed-Direct-Form-II), so it needs 60*n*_1_ multiplications and 27*n*_1_ additions in each *T* time period.

The memory requirement is *n*_1_ words for the BAC, *w*+*M*+*n*_2_ for the BACV and ABACV algorithms, only 59 for the FIR-based solution and 19 for the IIR-based algorithm.

In typical settings of the ABACV (*n*_1_ = 60, *n*_2_ = 400, *w* = 128, *M* = 128 and *ζ* = 0.02), the FIR-based (*n*_1_ = 90, and *O* = 59) and the IIR-based (*n*_1_ = 100) solutions require 274, 10,620 and 8,700 arithmetic operations (multiplications and additions) in each *T* time period, while the memory requirements are 656, 118 and 19 words for ABACV, the FIR-based and the IIR-based algorithm, respectively.

## 6. Summary

In this paper, a novel method was proposed to provide energy-efficient accelerometer-based event detection. An autocovariance-based signal processing algorithm was utilized to allow robust event detection, even in the case of a poor signal-to-noise ratio. The proposed solution allows a highly efficient implementation with very low computational needs; thus, the proposed algorithm can be implemented on low-end devices. The power consumption of the proposed method, used in a vehicle detection application, was reduced by a factor of five, with respect to the on-line version of the algorithm, while the error rate is very low. The proposed method outperformed the filter-based conventional solution in almost every respect: the proposed method has superior performance, lower power consumption and lower computational needs. The memory requirements, however, are higher than that of the filter-based solution, but still acceptable, even in low-end devices.

## Figures and Tables

**Table 1.** Performance comparison of the algorithms. RPC, relative power consumption; FP, false positive; FN, false negative.

		**Parameter Values**	**RPC [%]**	**FP [%]**	**FN [%]**
On-line algorithm [6]			100	0	0

BAC	*N*, *n*_1_, Θ	200, 50, 200	25	2.36	0
		300, 50, 200	16.67	1.58	0
		200, 70, 270	35	0.02	0.19
		300, 70, 270	23.33	0.01	1.3
		300, 60, 270	20	0.04	2.38

BACV	*N*, *n*_1_,*n*_2_, Θ	200, 50, 400, 50,000	37.33	0	0
		200, 50, 400, 80,000	37.33	0	0.63
		300, 50, 400, 70,000	26.54	0	0
		300, 55, 300, 70,000	25.56	0	1.25
		300, 50, 400, 35,000	26.54	0	0
		300, 50, 400, 30,000	26.54	0.04	0

ABACV	*N*, *n*_1_, *n_2_*,*ζ*,*λ*	200, 55, 400, 0.02, 8,	30.63	0.88	0
		200, 60, 400, 0.02, 8,	33.02	0	0
		300, 55, 400, 0.02, 8	20.86	0	0
		300, 55, 400, 0.02, 9,	20.86	0	0
		300, 55, 400, 0.02, 10,	20.86	0	0.84
		300, 55, 400, 0.03, 8,	21.82	0	0
		300, 55, 400, 0.02, 8	20.86	0	0
		300, 55, 400, 0.01, 8	19.86	0	1.25
		300, 45, 400, 0.03, 8	18.65	0	0.42

FIR	*N*, *n*_1_, *O*, Θ	200, 70, 59, 20,000	35	0.48	0.63
		200, 90, 59, 30,000	45	0.05	0
		300, 70, 59, 10,000	23.4	43.35	0.42
		300, 70, 59, 18,000	23.4	0.93	2.08
		300, 90, 59, 25,000	30	0.25	0.42
		300, 90, 30, 25,000	30	2.55	0
		300, 90, 30, 30,000	30	0.85	0.42
		300, 90, 15, 30,000	30	0.04	0.83

IIR	*N*, *n*_1_, Θ	200, 50, 40	25	0.13	31.25
		200,70, 190	35	0.16	15
		200, 100, 350	50	0.27	0.63
		200, 100, 300	50	0.73	0
		200, 100, 380	50	0.14	1.25
		200, 60, 90	30	0.38	18.75
		200, 60, 95	30	0.20	21.25
		200, 60, 85	30	0.73	15.63

## Appendix

### A. The Pseudo Code of the BAC Algorithm

**remark** *f_s_* = sampling frequency
*n*_1_ = ⌊*τ*_1_*f*_s_⌋
**procedure** BAC
**while** (**true**)
collect *n*_1_ samples into buffer **x**
compute *Ĉ_x_,_x_* using Equation (7)
**if** *Ĉ_x,x_* > Θ **then**
alarm on
**end**
sleep(*T* − *τ*_1_)
**end**
**end**

### B. The Pseudo Code of the BACV and ABACV Algorithms

**remark** *f_s_* = sampling frequency	
*n*_1_ = ⌊*τ*_1_*f*_s_⌋	
*n*_2_ = ⌊L*τ*_2_*f_s_*⌋	
**procedure** BACV/ABACV	
**repeat**
sleepTime :=*T* − *τ*_1_
collect *n*_1_ samples into buffer **x**
compute *Ĉ_x_,_x_* using Equation (7)
compute *trigger* using Equation (9)
**if** *trigger* = 1 **then**
collect and append *n*_2_ − *n*_1_ samples to buffer **x**
compute *c*(*k*), (*k* = 1… *n*_2_ − *w*) using Equation (10)
**if** *max_k_*(*c*(*k*)) > Θ **then**
alarm on
**end**
sleepTime := max {*T* − *τ*_2_, 0 }
**end**
**ADAPTATION** (**ABACV only**)
*rate* := *αrate* + (1 − *α*)*trigger*
*ϑ = ϑ* + *εsignum*(*rate* − *ζ*)
**if** *Ĉ_x,x_* < Σ **then**
$d=:d+(1-\alpha ){\hat{C}}_{x,x}^{2}$
end
Θ = λ*d*
**end of ADAPTATION**
sleep(sleepTime)
**end**
**end**
